# The Structure of *Treponema pallidum* Tp0751 (Pallilysin) Reveals a Non-canonical Lipocalin Fold That Mediates Adhesion to Extracellular Matrix Components and Interactions with Host Cells

**DOI:** 10.1371/journal.ppat.1005919

**Published:** 2016-09-28

**Authors:** Michelle L. Parker, Simon Houston, Helena Pětrošová, Karen V. Lithgow, Rebecca Hof, Charmaine Wetherell, Wei-Chien Kao, Yi-Pin Lin, Tara J. Moriarty, Rhodaba Ebady, Caroline E. Cameron, Martin J. Boulanger

**Affiliations:** 1 Department of Biochemistry and Microbiology, University of Victoria, Victoria, British Columbia, Canada; 2 Matrix Dynamics Group, Faculty of Dentistry, Department of Laboratory Medicine and Pathobiology, Faculty of Medicine, University of Toronto, Toronto, Ontario, Canada; 3 Department of Molecular Biology and Microbiology, Tufts University School of Medicine, Boston, Massachusetts, United States of America; University of Montana, UNITED STATES

## Abstract

Syphilis is a chronic disease caused by the bacterium *Treponema pallidum* subsp. *pallidum*. *Treponema pallidum* disseminates widely throughout the host and extravasates from the vasculature, a process that is at least partially dependent upon the ability of *T*. *pallidum* to interact with host extracellular matrix (ECM) components. Defining the molecular basis for the interaction between *T*. *pallidum* and the host is complicated by the intractability of *T*. *pallidum* to *in vitro* culturing and genetic manipulation. Correspondingly, few *T*. *pallidum* proteins have been identified that interact directly with host components. Of these, Tp0751 (also known as pallilysin) displays a propensity to interact with the ECM, although the underlying mechanism of these interactions remains unknown. Towards establishing the molecular mechanism of Tp0751-host ECM attachment, we first determined the crystal structure of Tp0751 to a resolution of 2.15 Å using selenomethionine phasing. Structural analysis revealed an eight-stranded beta-barrel with a profile of short conserved regions consistent with a non-canonical lipocalin fold. Using a library of native and scrambled peptides representing the full Tp0751 sequence, we next identified a subset of peptides that showed statistically significant and dose-dependent interactions with the ECM components fibrinogen, fibronectin, collagen I, and collagen IV. Intriguingly, each ECM-interacting peptide mapped to the lipocalin domain. To assess the potential of these ECM-coordinating peptides to inhibit adhesion of bacteria to host cells, we engineered an adherence-deficient strain of the spirochete *Borrelia burgdorferi* to heterologously express Tp0751. This engineered strain displayed Tp0751 on its surface and exhibited a Tp0751-dependent gain-of-function in adhering to human umbilical vein endothelial cells that was inhibited in the presence of one of the ECM-interacting peptides (p10). Overall, these data provide the first structural insight into the mechanisms of Tp0751-host interactions, which are dependent on the protein’s lipocalin fold.

## Author Summary

The *Treponema pallidum* protein, Tp0751, possesses adhesive properties and has been previously reported to mediate attachment to the host extracellular matrix components laminin, fibronectin, and fibrinogen. Herein we demonstrate that Tp0751 adopts an eight-stranded beta barrel-containing lipocalin structure, and using a peptide library approach we show that the extracellular matrix component adhesive functionality of Tp0751 is localized to the lipocalin domain. Further, using a heterologous expression system we demonstrate that Tp0751 mediates attachment to endothelial cells, and that this interaction is specifically inhibited by a peptide derived from the Tp0751 lipocalin domain. Through these studies we have delineated the regions of the Tp0751 protein that mediate interaction with host extracellular matrix components and endothelial cells. These findings enhance our understanding of the role of this protein in treponemal dissemination via the bloodstream and provide defined regions of the Tp0751 protein that can be targeted to disrupt the treponemal-host interaction.

## Introduction

Syphilis is a chronic, multistage disease caused by *Treponema pallidum* subsp. *pallidum*, with a global burden of 36 million cases and 11 million new infections per year [1]. Syphilis remains prevalent in resource-poor settings and the incidence rate is rising in Europe and Britain, the United States, Canada and China [2–7]. Congenital syphilis is the most common infection associated with fetal loss or stillbirth in low-income populations, with approximately 1.4 million pregnant women infected with active syphilis per year [8–10]. Symptomatic syphilis infections increase HIV transmission and acquisition 2- to 5-fold, and modeling studies predict that eradication of syphilis would have a significant impact on HIV prevention [11, 12]. Elimination of syphilis as a risk factor for HIV can be achieved only through prevention of new syphilis cases, since the highest risk for transmitting and acquiring HIV coincides with early syphilis and frequently precedes diagnosis. The continuing high rates and global public health threat of syphilis, despite the effectiveness of penicillin treatment, highlights the need for enhanced understanding of the mechanisms of *T*. *pallidum* pathogenesis.

The highly invasive nature of *T*. *pallidum* is reflected in its ability to cross the placental barrier (congenital syphilis), to invade the central nervous system (neurosyphilis), to cause a widespread rash (characteristic of secondary syphilis), and to invade immunologically privileged sites such as the eye (ocular syphilis) [13, 14]. Animal studies suggest dissemination via the bloodstream and lymphatics begins within hours of infection [15, 16], and early involvement of the liver and kidneys in patients implies that systemic dissemination is also an early event in humans [17, 18]. Although the invasive capability of *T*. *pallidum* is crucial to the pathogenesis of this microorganism, the molecular mechanisms underlying dissemination are incompletely understood. This is due, in part, to the fact that only a limited number of *T*. *pallidum* proteins have been identified that could be directly involved in molecular interactions with the host. Our understanding of the mechanisms underlying *T*. *pallidum* pathogenesis, and of dissemination in particular, is also limited by the inability to genetically modify this pathogen, and the associated challenges of studying the roles of candidate virulence factors in pathogenesis. Heterologous expression of candidate *T*. *pallidum* virulence factors in other spirochetes, including *Treponema phagedenis* [19] and more recently the Lyme disease spirochete *Borrelia burgdorferi* [20], is a crucial strategy for investigating the biological function of these factors.

One protein suggested to play a role in *T*. *pallidum* dissemination within the host is Tp0751 (also referred to as pallilysin). This protein is a primary target of opsonic antibodies, and thus is predicted to be surface-exposed on *T*. *pallidum*, and it binds and degrades host components encountered by *T*. *pallidum* during dissemination [21–25]. In particular, Tp0751 has a propensity to bind host molecules that are in close proximity to the vasculature, including ECM components found within the sub-endothelial matrix (laminin), and associated with the glycocalyx on the apical surface of endothelial cells (fibronectin and fibrinogen). Moreover, previous investigations using synthetic peptides identified the regions of Tp0751 that directly interact with laminin [22]. Although the functional studies performed to date indicate that Tp0751 interacts with multiple host components, the molecular basis for these interactions are unknown. A complicating factor is that the amino acid sequence of Tp0751 offers little insight into the molecular architecture and associated functions of the protein.

Towards establishing the molecular mechanisms by which Tp0751 engages host components, we first determined the three dimensional structure of the mature region of Tp0751 encompassing residues Ser78 to Pro237. Intriguingly, structural analysis revealed a beta-barrel fold displaying key hallmarks of a non-canonical lipocalin domain. Using a synthetic peptide library, we identified several peptides with the capacity to coordinate host ECM components and identified one of these peptides as possessing the ability to reduce adhesion of an engineered Tp0751-expressing *B*. *burgdorferi* strain to endothelial cells. Collectively, these data provide the first structural and mechanistic insight into Tp0751 interactions with host components.

## Results

### The C-terminal domain of Tp0751 adopts a compact beta-barrel conformation

**Fig 1:**
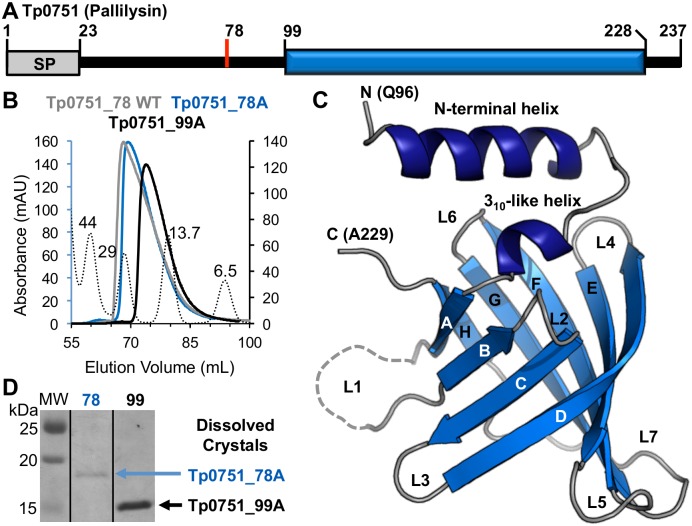
Tp0751 adopts a compact beta-barrel structure. **(A)** Schematic of the Tp0751 primary structure, indicating signal peptide (SP, grey box), putative thrombin cleavage site (Ser78, red line), and the region with predicted secondary structure elements (Val99 to His228, blue box). **(B)** Size exclusion chromatograms for constructs of Tp0751 (Tp0751_78A, 18 kDa; Tp0751_99A, 16 kDa) and globular standards (dotted lines: molecular weight (MW) in kDa listed above each peak) using a Superdex 75 HiLoad column. **(C)** Tertiary structure of Tp0751_78A with helices in dark blue, beta-strands in marine blue, and coils in grey, revealing an eight-stranded antiparallel beta-barrel capped by two helices. Note that the most N-terminal residue modeled is Gln96. **(D)** To assess if the N-terminal region (residues 78 to 95) was disordered in the crystal or proteolyzed, crystals were rinsed and loaded on an SDS-PAGE gel, revealing that the complete Tp0751_78A (predicted MW of 18 kDa) construct is packed in the crystal.

Analysis of the Tp0751 sequence C-terminal to the signal peptide cleavage site (Cys24 to Pro237) suggested a prolonged region of disorder extending to Pro98, followed by a set of defined secondary structure elements encompassing Val99 to His228 (Fig 1A). However, the lack of any significant sequence identity with known domains or structurally characterized proteins offered little insight into the architecture, and therefore function, of the C-terminal region of Tp0751. To address this knowledge gap, we first generated constructs for recombinant protein production that extended from Ser78 (putative thrombin cleavage site) or Val99 (beginning of region of predicted secondary structure) to the C-terminus (Tp0751_78 and Tp0751_99, respectively; Fig 1A). We also mutated Glu199 to Ala to stabilize the protein for crystallization studies [23]. Recombinant proteins produced in *E*. *coli* were purified and showed expected elution patterns using size exclusion chromatography, with the similar elution profiles of Tp0751_78 WT and Tp0751_78 E199A (Tp0751_78A) indicating that the point mutation did not alter protein folding (Fig 1B). We next crystallized and determined the three dimensional structure of Tp0751_78A using selenium single wavelength anomalous dispersion (SAD) phasing to a resolution of 2.15 Å (Table 1). Structural analysis revealed that the C-terminal domain of Tp0751_78A adopts a compact eight-stranded antiparallel beta-barrel with +1 topology, capped by a short 3_10_-like helix and a longer N-terminal helix (Fig 1C). The first ordered residue in the Tp0751_78A structure was Gln96 indicating the N-terminal residues Ser78 to Thr95 were either disordered or proteolyzed in the crystal. To investigate these two possibilities, crystals of Tp0751_78A, and the related Tp0751_99A construct for comparison, were isolated, washed and analyzed by SDS-PAGE. Analysis clearly revealed an intact N-terminal extension present in the Tp0751_78A crystals (Fig 1D) indicating that the lack of electron density for the N-terminal residues in the structure reflects disorder and not proteolysis. This confirms that the region of Tp0751 from Gln96 to Ala229 represents the core structural domain of the protein. In addition, an extended N-terminus on the Tp0751_78A construct is consistent with its faster elution from the size exclusion column compared to the globular protein standards (Fig 1B).

**Table 1:** Data collection and refinement statistics.

	Tp0751_78A-SeMet SAD	Tp0751_78A
A. Data collection statistics		
Spacegroup	H32	P3_1_21
a, b, c (Å)	146.05, 146.05, 152.82	144.72, 144.72, 152.61
α, β, γ (deg.)	90, 90, 120	90, 90, 120
Wavelength	0.9794	0.984
Resolution range (Å)	65.40–2.80 (2.95–2.80)	72.36–2.15 (2.19–2.15)
Measured reflections	338,530 (49,052)	754,198 (36,139)
Unique reflections	15,592 (2,245)	100,394 (4,902)
Redundancy	21.7 (21.8)	7.5 (7.4)
Completeness (%)	99.8 (100.0)	99.9 (100.0)
*I/σ(I)*	12.5 (2.9)	10.7 (2.7)
R_merge_	0.156 (0.966)	0.122 (0.719)
B. Refinement statistics		
Resolution (Å)		65.20–2.15
R_work_ / R_free_		0.239/0.276
No. of atoms		
Protein (A/B/C/D/		1039/1016/994/1034
E/F/G/H/I)		1029/1003/950/969/949
Solvent		188
Average B-values (Å^2^)		
Protein (A/B/C/D/		38.3/42.4/38.8/44.2
E/F/G/H/I)		41.1/45.0/52.1/48.1/51.9
Solvent		35.8
r.m.s. deviation from ideality		
Bond lengths (Å)		0.003
Bond angles (deg.)		0.642
Ramachandran statistics (%)		
Most favoured		93.4
Allowed		6.6
Disallowed		0.0

Values in parentheses are for the highest resolution shell

### Tp0751 adopts a non-canonical lipocalin fold

**Fig 2:**
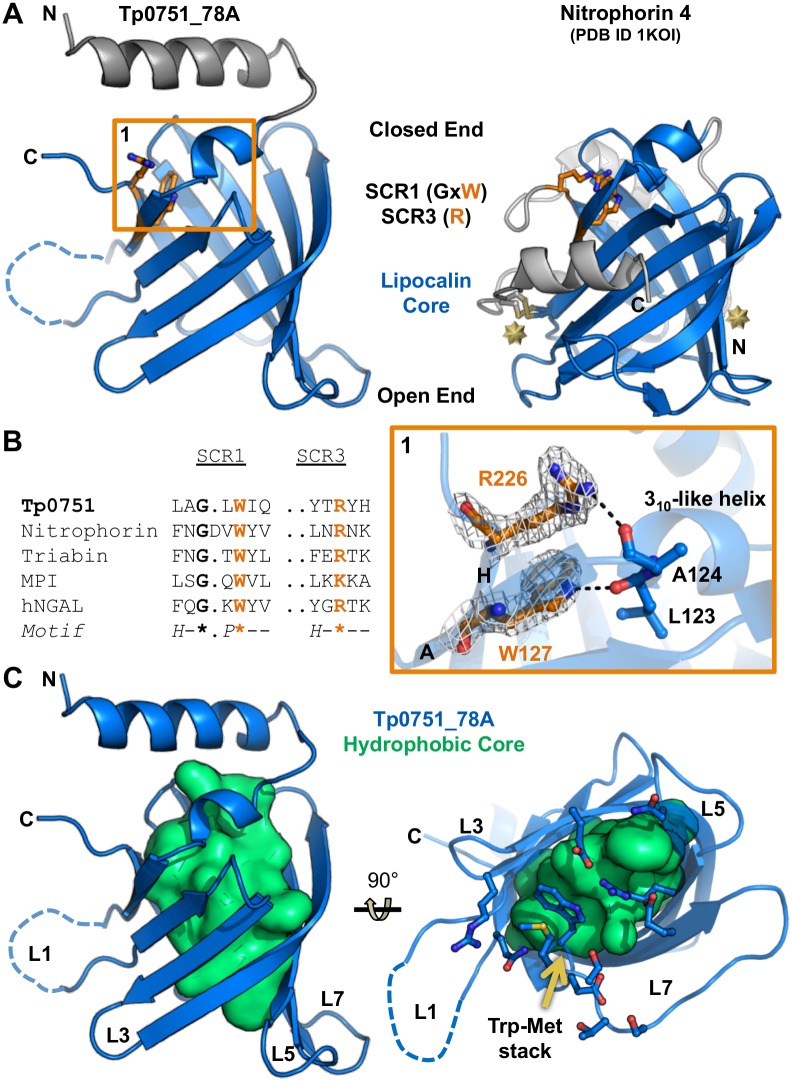
The structural domain of Tp0751 is an outlier lipocalin that lacks a readily accessible ligand binding cleft. **(A)** Left—Tertiary structure of Tp0751_78A with the lipocalin core colored marine and the unique N-terminal helix grey. Key residues of SCR1 and SCR3 are colored orange with side chains shown as sticks and highlighted by an orange box. Right—Nitrophorin 4 (PDB ID 4KOI) shown in the same orientation and color scheme as Tp0751_78A. N- and C-terminal disulfide bonds are shown as sticks and highlighted by gold starbursts. **(B)** Left—SCR1 and SCR3 sequences from Tp0751 and other lipocalins: Nitrophorin 4 (PDB ID 1KOI), Triabin (PDB ID 1AVG), MPI (metalloproteinase inhibitor, PDB ID 1SMP), and hNGAL (Human neutrophil gelatinase-associated lipocalin, PDB ID 1NGL). Key conserved residues are colored orange. For the motif, H is hydrophobic and P is polar. Right—Sigma-A weighted 2Fo-Fc electron density map contoured at 1.2 sigma around the SCR1 and SCR3 motif Trp127 and Arg226 residues of Tp0751_78A. Dashed lines indicate 2.8 Å hydrogen bonds. (**C**) Orthogonal views of the Tp0751_78A hydrophobic core (lime green). Residues that stack on the hydrophobic core at the open end are shown as sticks in the right panel with the Trp-Met stack anchoring Loop 7 indicated by the gold arrow. Note the lack of a pocket in the open end.

Comparison of the Tp0751_78A structure against the database of known structures using the DALI server [26] revealed a striking similarity to lipocalin domains, specifically nitrophorins. Tp0751_78A achieved a Z-score of 8.8 (Z < 2 is spurious) with the top hit Nitrophorin 4 (PDB ID 1KOI), corresponding to a root mean square deviation of 3.0 Å over 184 aligned positions (Fig 2A). Although Tp0751_78A and Nitrophorin 4 share only 6% sequence identity, low sequence identity is a common feature of lipocalins [27, 28]. Lipocalins, along with fatty acid-binding proteins and avidins, are members of the calycin superfamily, which is defined by the distinct features of a central beta-barrel and a key structural signature consisting of three short conserved regions (SCR1, SCR2, and SCR3) [27]. The designation of the Tp0751 structural domain as a lipocalin within the calycin superfamily is confirmed by the presence of eight antiparallel beta-strands with +1 topology that comprise the beta-barrel, combined with the elliptical shape of the barrel cross-section and readily distinguishable open and closed barrel ends (Fig 2A). While Tp0751_78A lacks certain features common to lipocalins, such as N- and C-terminal regions that are pinned to the outside of the beta-barrel by disulfide bonds, these are not requirements for classification as a lipocalin (Fig 2A, right). Ultimately, the classification of Tp0751 as an outlier lipocalin domain, as opposed to a kernel lipocalin, is based on the observation that is does not contain all three SCRs. This is also the basis for Nitrophorins being classified as outlier lipocalins [27]. The SCR1 in Tp0751 localizes to the 3_10_ helix and Strand A, centered on a GxW motif (Gly125 and Trp127 in Tp0751; Fig 2B) [27]. Although SCR3 is distal in amino acid sequence, residing on Strand H, the key basic residue in this region (Arg226 in Tp0751) stacks on top of the SCR1 Trp and forms a hydrogen bond to the backbone carbonyl of the preceding coil (Fig 2B) [27]. However, similar to other bacterial lipocalins [27], the SCR2, which is localized to the Strand F-Loop 6-Strand G region, is not conserved in Tp0751. Notably, this region was predicted previously [24] to incorporate a metal binding motif, however, the structure of Tp0751_78A reported here reveals no clear mechanism for metal coordination.

Lipocalins that are closely structurally related tend to have similar functions [28]. However, it is highly unlikely that Tp0751 shares a similar function with nitrophorins, as the residues required for heme coordination and transport in nitrophorins are not conserved in Tp0751. More broadly, a common feature of lipocalins is the presence of a hydrophobic ligand-binding pocket within the beta-barrel [27, 29]. This pocket and surrounding loops of the open end (L1, L3, L5, and L7) serve as a cup to coordinate an extensive variety of hydrophobic ligands for transport, catalytic, or sequestration purposes. In conventional lipocalins, Loop 1 serves as a lid for the cup-like binding site. Although Loop 1 of Tp0751_78A is exposed and partially disordered in the structure (Fig 2C), there is no discernable hydrophobic pocket, primarily because Loop 7 caps the hydrophobic core with a stacked Trp-Met pair (Fig 2C, right). While displacement of Loop 7 could expose a potential ligand-binding site, several other polar residues also cap the hydrophobic core (Fig 2C, right), suggesting that the open end of the Tp0751 lipocalin domain lacks a hydrophobic binding pocket.

### Tp0751 host ECM-binding peptides map to the lipocalin domain

**Fig 3:**
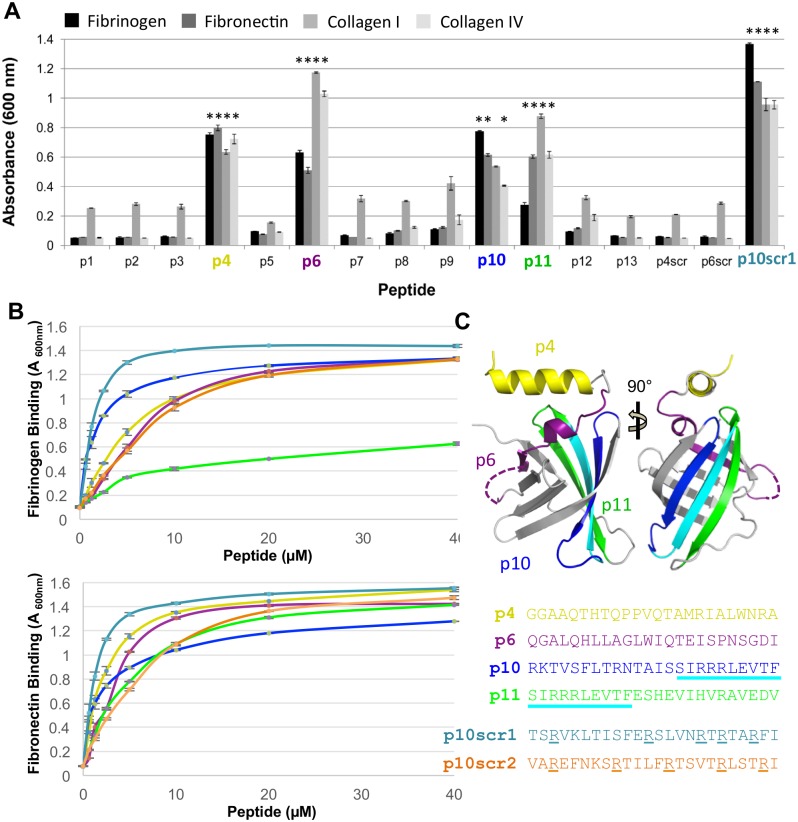
Tp0751 lipocalin derived peptides directly bind host proteins. **(A)** Thirteen overlapping Tp0751 synthetic 24-mer peptides were evaluated for binding to immobilized fibrinogen, fibronectin, collagen type I, and collagen type IV. Average absorbance readings (600 nm) from three wells are presented with bars indicating standard error and the results are representative of two independent experiments. For statistical analyses, attachment of each ECM-binding Tp0751 peptide (p4, p6, p10, p11 and p10scr1) to each host protein was individually compared to the level of binding individually exhibited by peptides p1, p2, p3, p5, p7, p8, p9, p12, p13, p4scr and p6scr to each ECM component, using the Student’s two-tailed *t*-test. Tp0751 peptides p4, p6, p11, and exhibited statistically significant levels of binding to all four host proteins (*p≤0.0113). Peptide p10 showed significant binding to fibrinogen, fibronectin, and collagen IV (*p*≤0.0006) but not to collagen I. **(B)** Dose-dependent binding assays with Tp0751 peptides p4, p6, p10, p11, p10scr1, and p10scr2 to fibrinogen (Fg—upper panel) and fibronectin (Fn—lower panel). Average absorbance readings (600 nm) from three wells are presented with bars indicating standard error and the results are representative of two independent experiments. Apparent *K*_d_ values, calculated using GraphPad Prism, were as follows: p4 = 6.9 ± 0.4 μM (Fg) / 2.4 ± 0.1 μM (Fn); p6 = 10.5 ± 2.0 μM (Fg) / 4.5 ± 0.9 μM (Fn); p10 = 1.6 ± 0.1 μM (Fg) / 1.6 ± 0.2 μM (Fn); p11 = 9.2 ± 1.0 μM (Fg) / 5.8 ± 0.3 μM (Fn); p10scr = 1.5 ± 0.2 μM (Fg) / 1.3 ± 0.1 μM (Fn), and p10scr2 = 11.0 ± 1.3 μM (Fg) / 7.9 ± 0.7 μM (Fn) **(C)** Peptides p4 (yellow), p6 (purple), p10 (blue/cyan) and p11 (cyan/green) are mapped onto the Tp0751 lipocalin domain. The strand colored in cyan reflects the overlap of p10 and p11. Color coded sequences of peptides p4, p6, p10, p10scr1, and p10scr2 are shown. Arginine residues in the p10 peptides are indicated by underlining.

To dissect the contributions of individual substructures in mediating attachment to host ECM components, we took advantage of a Tp0751 peptide library [22]. Of the 13 native Tp0751 peptides tested (p1-p13) in our initial binding screen, p4, p6, and p11 displayed statistically significant binding to fibrinogen (*p*≤0.0004), fibronectin (*p*≤0.0001), collagen I (*p*≤0.0113), and collagen IV (*p*≤0.0002) (Fig 3A). Peptide p10 also showed significant binding to fibrinogen (*p<*0.0001), fibronectin (*p*<0.0001), and collagen IV (*p*≤0.0006), but not to collagen I (*p*≤0.0653). The overlapping peptides 3, 5 and 7 exhibited little to no binding, consistent with a key role for the central four amino acids unique to p4 (PVQT) and p6 (LWIQ) in mediating interactions. Notably, scrambled versions of p4 (p4scr) and p6 (p6scr) showed no binding, yet a scrambled p10 (p10scr1) showed enhanced binding relative to p10 (Fig 3A).

To further investigate the strength and specificity of these interactions, we performed dose-dependent binding assays. First we measured the apparent dissociation constants (*K*_d_) of Tp0751_78A (2.0 ± 0.4 μM) and Tp0751_78 WT (6.1 ± 1.7 μM) to fibrinogen (S1 Fig). The tighter binding observed with Tp0751_78A is likely due to its greater stability as observed previously [23]. Next we showed that the Tp0751 peptides p4, p6, p10, and p11 exhibited dose-dependent binding to both fibrinogen and fibronectin with apparent dissociation constants ranging from 1.6 to 10.5 μM (see Fig 3B legend for individual apparent *K*_d_ values). To further probe the previously observed enhanced binding with p10scr1 in the single point assay, we measured dose dependent binding and calculated corresponding apparent *K*_d_ values of 1.5 ± 0.2 μM and 1.3 ± 0.1 μM to fibrinogen and fibronectin, respectively (Fig 3B). Analysis of p10 sequence revealed an arginine triplet framed by hydrophobic residues (Fig 3C) that remained largely intact in p10scr1 (RxRxxR). To test the contribution of this Arg rich region, we generated a second scrambled version of p10 (p10scr2) in which the arginine residues are more spatially separated. Intriguingly, p10scr2 exhibited weaker binding to fibrinogen (apparent *K*_d_ = 11 ± 1.3 μM) and fibronectin (apparent *K*_d_ = 7.9 ± 0.7 μM) compared to p10scr1 and p10 (Fig 3B). Collectively, these data reveal a key, yet highly contextual role for the arginine motif in coordinating host proteins.

We next mapped each of the ECM binding peptides onto the Tp0751 structure. Notably, each peptide that exhibited binding is contained within the lipocalin domain, highlighting the importance of this ordered region for interfacing with the host (Fig 3C). Peptide 4 (yellow) maps to the N-terminal helix while p6 (purple) maps to the 3_10_-like helix and a short strand (Strand A labeled in Fig 1C) and peptides p10 (blue/cyan) and p11 (cyan/green) map to Strands E/F and F/G, respectively.

### Expression and surface localization of Tp0751 in *B*. *burgdorferi*

**Fig 4:**
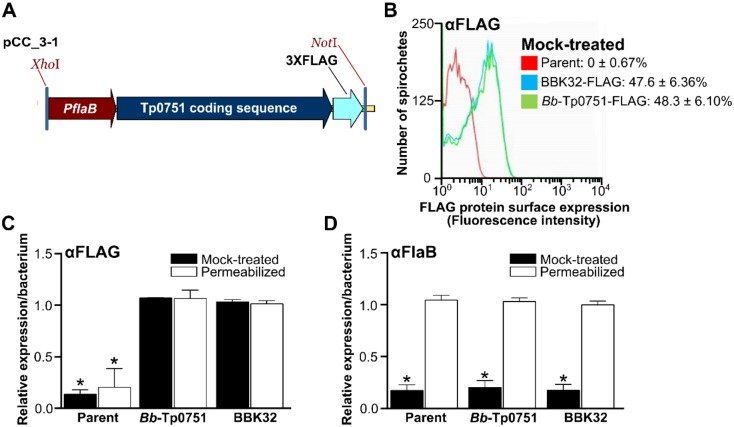
Expression and surface localization of Tp0751 in *B*. *burgdorferi*. **(A)** Schematic depicting Tp0751 expression construct in *B*. *burgdorferi* including 1) constitutive *flaB* promoter (P_*flaB*_), 2) Tp0751 lipoprotein localization signal sequence and Tp0751 coding sequence, and 3) a C-terminal 3X-FLAG tag. Inserts were cloned into XhoI and NotI sites of a pCE320 (*cp32*)-derived shuttle vector (resulting plasmid pCC_3–1), and transformed into a non-infectious, adhesion-attenuated B31-A-derived GFP-expressing parent strain (GCB706). Strain names and details are provided in S1 and S2 Tables. (**B-D**) Fluorescence-activated cell sorting (FACS) analysis of Tp0751 localization on the surface of *B*. *burgdorferi*. Strains were mock-treated with phosphate based saline buffer or permeabilized with methanol before probing with antibodies to the FLAG tag (part of BBK32 and Tp0751 fusion proteins), or to constitutively expressed periplasmic (non-surface-localized) flagellin B protein (FlaB). (**B**) Representative histogram depicting fluorescence intensities of indicated mock-treated *B*. *burgdorferi* strains probed with anti-FLAG antibodies. The mean ± SEM percentages of bacteria that were FLAG-positive for each strain in all replicates are indicated. (**C-D**) Relative expression levels per bacterium of FLAG-tagged fusion proteins Tp0751 and BBK32 (**C**) and FlaB (**D**) in mock-treated and methanol-permeabilized *B*. *burgdorferi*. Mean ± SEM fluorescence intensities for each strain were expressed as a proportion of mean fluorescence intensity for *B*. *burgdorferi* expressing BBK32-FLAG. N = 8 independent cultures per strain analyzed in two FACS experiments. Statistical analysis: One-way Kruskal-Wallis ANOVA with Dunn’s post-test. * indicates p<0.05 vs. permeabilized BBK32-FLAG control within treatment (mock or permeabilized).

We next sought to investigate the capacity of wild-type Tp0751 to attach to endothelial cells in the biologically relevant context of a live spirochete. Due to the technical limitations associated with direct experimentation with *T*. *pallidum*, we conducted these studies by engineering the model spirochete, *B*. *burgdorferi*, to heterologously express Tp0751 as a surface-localized protein (strain *Bb*-Tp0751). To enable these investigations, we cloned the *tp0751* open reading frame, including the Tp0751 lipoprotein localization signal sequence, in fusion with sequences encoding a C-terminal 3X-FLAG tag under the control of a constitutive *B*. *burgdorferi* promoter (*P*_*flaB*_) and inserted this construct into a cp32-based shuttle vector (Fig 4A). A construct for constitutive expression of C-terminally FLAG-tagged BBK32 (a *B*. *burgdorferi* adhesin [30, 31]) was also generated as a positive control. The resulting constructs were transformed into a non-infectious, BBK32-negative, adhesion-attenuated, GFP-expressing, B31-A-derived *B*. *burgdorferi* strain [30, 31]. Positive clones were screened by PCR with adhesin-encoding inserts sequence verified, and antibiotic selected (S2 Fig). Digital PCR also verified *tp0751* transcripts were produced in the *Bb*-Tp0751-transformed strain (S3 Fig).

To determine if Tp0751 was expressed and surface-localized on the heterologous expression strain, we measured expression of the C-terminal FLAG tag of fusion proteins and a non-surface-localized control protein (periplasmic flagellin B, FlaB) by fluorescence-activated cell sorting (FACS) analysis using antibodies to the FLAG tag and FlaB (Fig 4B–4D). FACS analysis was performed with equal numbers of methanol-permeabilized and mock saline-treated bacteria to distinguish between surface-localized and intracellular proteins. A representative histogram of mock saline-treated strains probed with anti-FLAG antibodies is shown in Fig 4B. Approximately 50% of bacteria transformed with plasmids encoding FLAG-tagged BBK32 and Tp0751 were FLAG-positive, with no significant differences among strains (p = 0.94), whereas 0% of parent strain bacteria were positive for the FLAG epitope (Fig 4B). This indicated that detection of surface-localized proteins via their FLAG tag was specific. Quantification of FLAG tag and FlaB expression levels in methanol-permeabilized and mock-treated bacteria showed that FLAG fusion proteins were exclusively localized to bacterial surfaces (Fig 4C: p≥0.66 mock vs permeabilized) and that periplasmic FlaB was not readily detected without permeabilization (Fig 4D). Thus, FACS analysis was performed under conditions that did not damage bacterial outer membranes and expose intracellular proteins. The BBK32- and Tp0751-expressing strains showed similar relative abundance of FLAG-tagged proteins and there was no difference in expression of FlaB (Fig 4C and 4D: p≥0.7 for all comparisons). Therefore, Tp0751 was expressed and surface-localized on *B*. *burgdorferi* as efficiently as the *B*. *burgdorferi* lipoprotein BBK32.

Although a constitutive *B*. *burgdorferi* promoter was used to drive expression of FLAG fusion proteins and FACS analysis showed that 50% of bacteria expressed these proteins (Fig 4B), we did not detect expression of Tp0751 by either immunoblotting or immunofluorescence. Although the reason for this discrepancy is unknown, we hypothesize that the capacity of FACS to facilitate analysis at the single-cell level, combined with the exquisite sensitivity of FACS [32], accounts for the divergent results obtained between FACS and the other immunological techniques.

### The host ECM binding peptide p10 prevents bacterial adhesion to host cells

**Fig 5:**
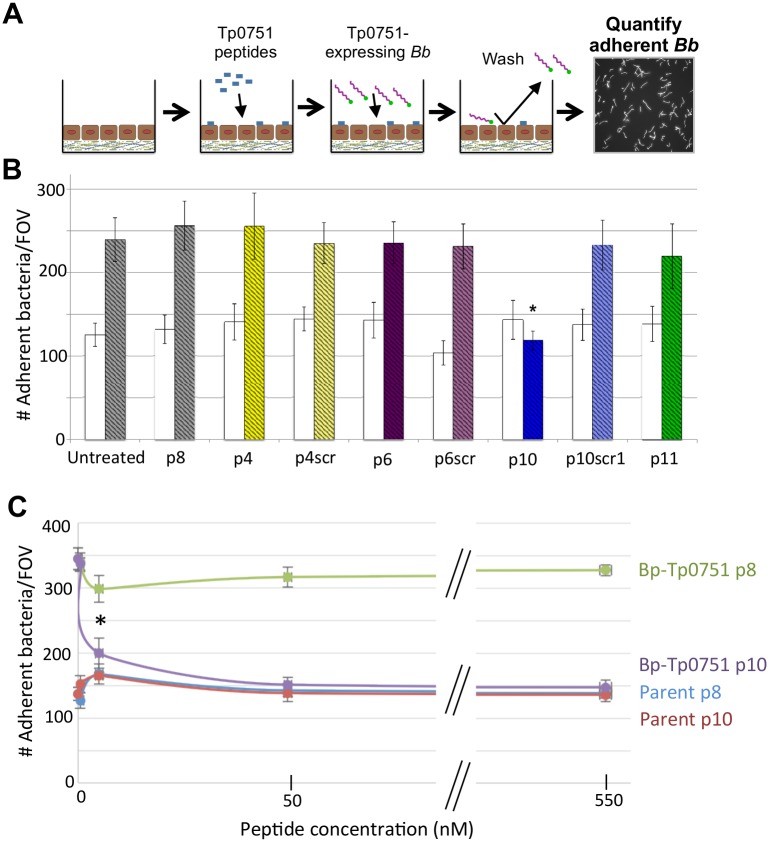
Tp0751 peptide 10 inhibits adhesion of Tp0751-expressing *B*. *burgdorferi* to HUVEC monolayers. **(A)** Schematic showing the experimental design of the competitive inhibition assay. Confluent HUVEC monolayers seeded on artificial basement membrane were preincubated with synthetic Tp0751 peptides (p4, p6, p10, p11, p4scr, p6scr, and p10scr1) to allow for peptide-endothelial cell interactions. HUVECs were then co-incubated with parent (negative control) or Tp0751-expressing *B*. *burgdorferi* strains to assess the competitive inhibition capacity of the synthetic Tp0751 peptides. After washing to remove non-adherent bacteria, *B*. *burgdorferi* adhesion to HUVECs was quantified using fluorescence microscopy to count the number of adherent bacteria per field of view (FOV) at 400x magnification. **(B)** Bar graph illustrating the number of adherent *B*. *burgdorferi* per FOV. Mean counts ± SEM from ten FOV for each biological replicate are presented with standard error bars. For statistical analyses, attachment by strain *Bb*-Tp0751 to HUVEC monolayers preincubated with peptides p4, p6, and p10 was compared to the attachment of strain *Bb*-Tp0751 in the presence of corresponding scrambled peptides, using the Student’s two-tailed *t*-test. Strain *Bb*-Tp0751 exhibited statistically significant lower levels of binding to HUVEC monolayers preincubated with peptide p10 (*p≤0.005) when compared to the levels of binding when preincubated with the scrambled version of peptide 10 (p10scr1). *Bb*-Tp0751 adhered significantly more to endothelia than the parent under untreated conditions and when monolayers were pre-incubated with all other peptides (p˂0.05). The parent and *Bb*-Tp0751 strains are indicated by clear white bars and striped colored bars, respectively. **(C)** Line graph representing the effect of increasing concentrations (0 nM, 0.54 nM, 5.45 nM, 54.5 nM, and 545 nM) of p10 and negative control peptide p8 on adherence of Parent and *Bb*-Tp0751 to HUVECs. Mean counts ± SEM from ten FOV for each biological replicate are presented with standard error bars. Statistical analysis, was performed using the Student’s two-tailed *t*-test. Strain *Bb*-Tp0751 exhibited significantly lower levels of binding to HUVEC monolayers when preincubated with ≥ 5.45 nM p10 compared to the levels of binding when preincubated with ≥ 5.45 nM p8 (*p≤0.005). A non-linear regression curve was fitted and the IC50 for p10 inhibition of HUVEC binding by *Bb*-Tp0751 was calculated (IC50 = 17 nM) using GraphPad Prism data analysis software (San Diego, CA).

With the knowledge that lipocalin-derived Tp0751-specific peptides bind host components, we next investigated the possibility that the Tp0751 ECM-binding peptides could modulate adhesion of our Tp0751-expressing *B*. *burgdorferi* (strain *Bb*-Tp0751) to host cells (Fig 5A). We compared the ability of *Bb*-Tp0751 to adhere to HUVECs (Human Umbilical Vein Endothelial Cells) relative to the non-adherent, non-transformed *B*. *burgdorferi* strain (Parent), alone or individually preincubated with the Tp0751 synthetic peptides. While peptides p4, p6, and p11 did not alter adhesion of *Bb*-Tp0751, incubation with peptide p10 significantly reduced binding of *Bb*-Tp0751 to HUVEC monolayers (*p*≤0.005) compared to the binding levels exhibited following preincubation with the scrambled peptide, p10scr1 (Fig 5B). A negative control peptide (p8) that failed to bind the ECM components tested in the current study (Fig 3A) had no effect on HUVEC adherence by either *B*. *burgdorferi* strain. Furthermore, preincubation of HUVEC cells with increasing concentrations of p10 (0 nM– 545 nM) resulted in dose-dependent and statistically significant lower levels of binding of *Bb*-Tp0751 to HUVEC monolayers compared to the levels of binding when preincubated with p8 (≥ 5.45 nM p10; *p*≤0.005). These results show that p10 is uniquely capable of inhibiting binding of Tp0751-expressing *B*. *burgdorferi* to HUVEC monolayers.

## Discussion

The ability to disseminate throughout the host and extravasate from the vasculature is central to the pathogenesis of the spirochete bacterium *T*. *pallidum*. As with other invasive pathogens, these processes in *T*. *pallidum* are undoubtedly governed by surface displayed proteins that enable adhesion to host components. The identification and characterization of *T*. *pallidum* adhesins, however, has proven particularly challenging due to the intractability of the pathogen to *in vitro* culturing, genetic manipulation and the use of conventional experimental methodologies. As a result, very few *T*. *pallidum* adhesins (Tp0136 [33, 34], Tp0155 [35], Tp0483 [35] and Tp0751 [23, 24]) have been identified. Of these, we have focused on Tp0751 due to its role in modulating host ECM interactions and because its targeting by opsonic antibodies in intact *T*. *pallidum* [24] indicates that it is surface-localized in this pathogen.

The structural, biochemical and functional analyses described herein reveal an intriguing profile for Tp0751 that relies on a compact, eight-stranded beta-barrel classified as a non-canonical or outlier lipocalin fold (Fig 1). The overall architecture of lipocalins supports diverse functional roles in both prokaryotes and eukaryotes, where the latter are localized to the extracellular milieu and implicated in binding cell surface receptors, regulation of cell homeostasis, and modulation of immune and inflammatory responses [36]. Eukaryotic lipocalins are also involved in modulating host cell signaling pathways that regulate cell motility and cell differentiation, and in this way have been reported to promote tumor metastasis [37–39]. In contrast, many of the earliest characterized prokaryotic lipocalins are localized to the inner leaflet of the outer membrane in Gram negative bacteria [40]. They rely on a central cavity to coordinate hydrophobic ligands, and function in outer membrane biogenesis and repair [40] and membrane adhesion [41]. More recently, however, an expanded functional repertoire for prokaryotic lipocalins has been recognized. These include the secreted lipocalin-containing protein Hp1286 from *Helicobacter pylori* that is involved in bacterial colonization and persistence in the stomach [41], and the recent report of lipocalin YxeF in the Gram-positive bacterium *Bacillus subtilis* [42]. Most importantly, the lipocalin domain of the factor H binding protein (fHbp) from *Neisseria meningitides* has been shown to be surface-localized and lack a hydrophobic pocket [43–45]. Intriguingly, our structural investigations of Tp0751 also reveal the absence of a defined hydrophobic binding pocket (Fig 2). This feature, combined with the prior observation that Tp0751 binds host ECM components [23, 24] and our observation reported here that Tp0751 is surface-localized when heterologously-expressed in the related spirochete *B*. *burgdorferi*, is consistent with a functional role for Tp0751 that centers on host-pathogen protein-protein interactions.

To further dissect the capacity of Tp0751 to coordinate host partners, we employed a peptide library that spanned the entire post-signal peptide Tp0751 sequence and showed that host ECM component binding was entirely contained within the lipocalin domain (Fig 3). Moreover, and in similar fashion to *N*. *meningitides* fHbp [43–45], much of the binding surface is localized to one face of the lipocalin beta-barrel and formed by peptides p10 and p11 (Fig 3). Intriguingly, the 10 amino acid overlapping region (Fig 3—cyan) between these peptides harbors an arginine triplet framed by hydrophobic residues. Consistent with the observation of an influential role for basic residues in host component attachment, it has been shown that the FbsA adhesin from *Streptococcus agalactiae* binds fibrinogen via a 16-amino acid motif containing RRxR/K and xxR/Kxx sequences [46]. Studies using site-directed mutagenesis and synthetic peptides have also shown positively charged residues to be important in mediating binding of different MSCRAMMs (Microbial Surface Components Recognizing Adhesive Matrix Molecules) to fibrinogen and fibronectin [47–49]. Furthermore, both arginine and lysine residues have been shown to comprise part of the fibronectin binding motif in BBK32 from *B*. *burgdorferi* [50]. While peptides 4 and 6 do not harbor a basic region, they also show significant binding to host ECM components. Thus, these data reveal a spatially and chemically diverse strategy employed by Tp0751 to coordinate host ECM partners that likely supports adhesion of *T*. *pallidum* to the vasculature and has the potential to serve as an immune-masking strategy [51].

To investigate the role of Tp0751 in mediating binding to the host endothelium, we engineered a *B*. *burgdorferi* strain to heterologously express and display Tp0751 on its surface (Fig 4). The expression of the *tp0751* gene within *B*. *burgdorferi* was verified at both the transcript and protein levels using digital PCR and FACS analyses, respectively (Figs 4 and S3). The detection of Tp0751 expression in intact *Borrelia*, combined with the detection of FlaB solely in permeabilized *Borrelia*, verifies expression of Tp0751 on the borrelial surface, thus placing Tp0751 in a location that is directly exposed to the host environment. Thus, our studies clearly show that the native Tp0751 lipoprotein localization peptide is sufficient for surface localization on *B*. *burgdorferi*. Notably, this observation suggests the existence of a conserved lipoprotein export system among diverse spirochetes and provides further evidence to suggest that Tp0751, (and potentially other lipoproteins), is a surface-localized lipoprotein in *T*. *pallidum*. With this powerful new model system, we then showed that p10 was uniquely capable of competitively inhibiting binding of the Tp0751-expressing *B*. *burgdorferi* to HUVEC monolayers (Fig 5) in a dose-dependent manner. Based on the important role of p10 we further investigated the structure of this peptide in the context of the lipocalin fold. Specifically, the 24mer sequence can be broken into four distinct sub-structures: L4 (RK), Strand E (TVSFLTRN), L5 (TAISS), and Strand F (IRRRLEVTF). In the context of the lipocalin fold, nearly all the hydrophobic residues of p10 are buried in the protein core, while the six basic residues are clustered predominately in two basic patches separated at the closed end (RK on L4) and near the open end (RRR on Strands E and F) of the lipocalin. However, the interaction with HUVECs appears to be not solely dependent on the arginine-rich region found in p10, since both p11 and the scrambled p10 peptides contained this positively charged region yet did not significantly inhibit binding. Thus, it appears that the capacity of Tp0751 to engage molecular partners on endothelial cells is mediated by a different, more narrowly defined mechanism, compared with those used in ECM coordination. The lack of a central hydrophobic pocket in Tp0751 suggests a protein-protein interaction function mediated by the extended surfaces of the compressed beta-barrel. Dissecting Tp0751 using peptide libraries and host cell component binding assays ultimately validated this hypothesis.

**Fig 6:**
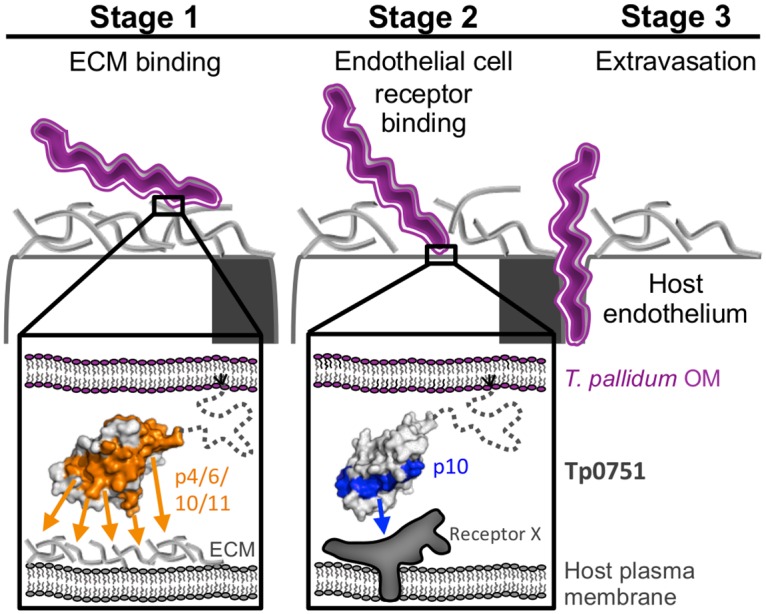
Three-stage model for *T*. *pallidum* binding host components and extravasation. Left–*T*. *pallidum* (purple bacterium) initially interacts with ECM components. Inset: Tp0751 (grey surface; dotted line represents a predicted disordered N-terminus embedded in the membrane by a lipid anchor) presented on the outer membrane (OM) mediates attachment to host endothelium via a large surface consisting of p4, p6, p10, and p11 (red surface) forming promiscuous contacts with ECM components. Middle—Defined adhesion between *T*. *pallidum* and endothelial cells mediated via a region of Tp0751 (inset) isolated to p10 (blue surface) and a specific, unidentified host cell receptor (Receptor X). Right—Transmigration of *T*. *pallidum* during extravasation.

To place these data in the context of *T*. *pallidum* dissemination and invasion, we propose a three stage model, with two stages for binding to host components, followed by a final stage of extravasation (Fig 6). In the first stage, the surface-exposed Tp0751 leverages its promiscuous binding surface (p4, 6, 10 and 11) to engage host ECM components as an effective mechanism of slowing bacterial movement through the vasculature. In the second stage, once the bacterium has slowed, Tp0751 engages specific host endothelial receptors using a more narrowly focussed molecular interaction strategy mediated solely by the region represented by p10. Such a multi-stage binding mechanism has been demonstrated to occur between *B*. *burgdorferi* and endothelial cells under conditions of flow, with BBK32 mediating initial interactions with ECM components followed by more specific interactions with endothelial surfaces [30]. We propose that a similar binding mechanism plays a key role in treponemal virulence, and that disrupting these interactions has the potential to limit infectivity of this invasive pathogen. Thus Tp0751, much like the *N*. *meningitides* fHBP surface-displayed lipocalin, which is one of three protein antigens included in the highly successful 4CMenB vaccine [52, 53], may be an ideal candidate for targeted vaccine development.

Collectively, our findings provide the first detailed structural and mechanistic insight into the molecular cross talk between *T*. *pallidum* and host cells and offer intriguing potential for developing Tp0751-based measures to control dissemination and, ultimately, pathogenesis of the syphilis spirochete.

## Materials and Methods

### Cloning, protein production and purification

Constructs encoding N-terminally truncated forms of Tp0751 (Ser78 to Pro237, Tp0751_78; Val99 to Pro237, Tp0751_99) with an E199A mutation as previously described [24] were cloned into a modified pET28a vector with a TEV protease cleavable N-terminal hexa-histidine tag. Constructs were produced in *E*. *coli* BL21 and purified from the soluble fraction by Ni-affinity chromatography. Tp0751_78A and Tp0751_99A were cleaved with TEV protease, purified further by size exclusion and cation exchange chromatography, and exchanged into a final buffer of 20 mM HEPES pH 8.0, 150 mM NaCl, 1% glycerol, and 5 μM zinc chloride. Selenomethionine (SeMet)-labelled Tp0751_78A protein was produced using *E*. *coli* 834 cells in SeMet media (Molecular Dimensions). The culture was grown at 37°C to an *A*_600_ of 1.2, cooled to 16°C and then induced at an *A*_600_ of 1.8 with 0.4 mM isopropyl 1-thio-β-d-galactopyranoside. After 18 h of growth at 16°C, the cells were harvested by centrifugation, and the SeMet-labeled protein was purified using the same protocol as for the native protein.

### Crystallization and data collection

Crystals of Tp0751_78A were originally identified in the SaltRx screen (Hampton Research) using sitting drops at 18°C. The final, refined drops consisted of 0.8 μL Tp0751_78A at 15 mg/mL with 0.8 μL of reservoir solution (0.1 M sodium acetate pH 5.4, 1.6 M sodium formate) and were equilibrated against 120 μL of reservoir solution. Crystals appeared within 5 days and grew to a final size within 3 weeks. For crystallization of SeMet-derivatized protein, 1.0 μL of Tp0751_78A-SeMet at 15 mg/mL was mixed with 1.0 μL of reservoir solution (0.1 M sodium acetate pH 3.6, 1.6 M sodium formate) and equilibrated against 120 μL of reservoir solution. Crystals were cryoprotected in paratone and flash cooled in liquid nitrogen. Diffraction data were collected on beamline 08ID-1 at Canadian Light Source using a wavelength of 0.984 Å for the native data, or an optimized wavelength of 0.9794 Å for the *f*” selenium edge.

### Data processing, structure determination and refinement

Diffraction data were processed to 2.15 Å (Tp0751_78A) or 2.80 Å (Tp0751_78A-SeMet) resolution using Imosflm [54] and Scala [55] or Aimless [56] in the CCP4 suite of programs [57]. The structure of Tp0751_78A-SeMet was solved by Selenium single wavelength anomalous dispersion. A total of 6 high confidence Se sites were identified using Phenix.hyss, and enabled building and registering of approximately 70% of the backbone using Phenix.autosol followed by Phenix.autobuild [58]. Native data were twinned; while Phenix.xtriage [58] and Aimless [56] identified P622 as the lattice point group, Zanuda in CCP4 [57] identified P321 as the most likely space group. The Tp0751_78A native structure was solved by molecular replacement using a single Tp0751_78A chain from the Se-phased model in Phaser [59], which identified P3_1_21 as the optimal space group. COOT [60] was used for model building and selection of solvent atoms, and the model was refined in Phenix.refine [61] using a twin fraction of 0.43 and the merohedral twin operator -H, -K, L. The structure of Tp0751_78A has 9 copies in the asymmetric unit; each chain overlays on chain A with an rmsd of 0.30 to 0.60 Å over 115 to 127 aligned Cα positions. Chain A was the most completely modeled with the lowest thermal motion parameters and was used for all analyses and figures. Complete structural validation was performed with Molprobity [62], including analysis of the Ramachandran plots, which showed greater than 93% of residues in the most favored conformations. Five percent of reflections were set aside for calculation of R_free_. Data collection and refinement statistics are presented in Table 1. The atomic coordinates and structure factors for Tp0751_78A have been deposited in the Protein Data Bank under the following PDB ID: 5JK2.

### Synthetic Tp0751 peptides

Thirteen overlapping 24-mer peptides (p1-p13) that spanned the Tp0751 sequence from T46-P237 were synthesized as described previously [22]. Each peptide shared 10 overlapping amino acids with neighboring upstream and downstream peptides. Scrambled versions of peptides 4 (p4scr), 6 (p6scr), 10 (p10scr1), and a second scrambled version of peptide 10 (p10scr2—VAREFNKSRTILFRTSVTRLSTRI) were prepared as described previously [22]. All synthetic peptides contained N-terminal hexa-histidine tags to allow for detection.

### Host proteins

Plasminogen-depleted human fibrinogen (Calbiochem) was purchased from VWR International. Laminin isolated from Engelbreth-Holm-Swarm murine sarcoma basement membrane and fibronectin isolated from human plasma were purchased from Sigma-Aldrich Canada Ltd. (Oakville, ON). Human collagen types I and IV (Rockland Immunochemicals, Inc.) were purchased from VWR International.

### *In vitro* host protein binding assays

To test for binding of synthetic Tp0751 peptides p1-p13, p1scr, p6scr, p10scr1, and p10scr2 to the host proteins fibrinogen, fibronectin, laminin, collagen type I, and collagen type IV, initial binding assays were performed as described previously [21, 22]. For dose-dependent binding assays, recombinant proteins were serially diluted 1:2 from either 10 μM to 0.156 μm (Tp0751_78A used for crystallization and Tp0751_78 WT) or 40 μm to 0.625 μm (peptides p4, p6, p10, p11, p10scr1, and p10scr2). Average absorbance readings (600 nm) from three wells are presented with bars indicating standard error and the results are representative of two independent experiments. Plates were read at 600 nm with a BioTek enzyme-linked immunosorbent assay plate reader (Fisher Scientific, Ottawa, ON). All statistical analyses were performed using the Student’s two-tailed *t*-test. Non-linear regression curves were fitted and apparent dissociation constants (*K*_d_) were calculated using GraphPad Prism data analysis software (San Diego, CA).

### *Borrelia burgdorferi* strains and growth conditions

All bacterial strain details are provided in S1 Table. Construction and characterization of GFP-expressing the non-infectious B31-A-derived parent (GCB706) strain was previously reported [63]. Construction and characterization of the GCB706-derived BBK32-3XFLAG- (TMB103) and Tp0751-3XFLAG-expressing (designated TMB49 or *Bb*-Tp0751) strains are described below and in S2 Table. *B*. *burgdorferi* was cultivated as reported [63] in Barbour-Stoenner-Kelly-II (BSK-II) medium supplemented with 100 μg/ml gentamicin and/or 200 μg/ml kanamycin for plasmid selection.

### Construct cloning for heterologous expression

All primers and templates used for construct cloning, *E*. *coli* construct names and strain numbers, and *B*. *burgdorferi* strain names are described in S1–S3 Tables. Constructs for expression of C-terminally 3XFLAG-tagged BBK32 (pTM259) and Tp0751 (pCC_3–1) were assembled by overlap extension PCR, cloned into pJET1.2 by blunt ligation, sequenced using pJet1.2 forward and reverse primers (Fermentas, Burlington, ON), excised and cloned into XhoI/NotI sites of cp32-derived shuttle vector pCE320 as described [64], followed by sequencing with primers B1723 and B1724. *E*. *coli* strain TOP10 (Life Technologies, Burlington, ON) was used for cloning Tp0751 expression cassettes into pJET1.2. *E*. *coli* DH5α was used for all other cloning steps and preparation of plasmid for *B*. *burgdorferi* transformations. *P*lasmids were prepared using Qiagen maxiprep kits (Qiagen, Toronto, ON).

### *B*. *burgdorferi* transformations

Electrocompetent GCB706 *B*. *burgdorferi* were prepared and transformed with 50 μg pTM259 or pCC_3–1 as described [30], followed by cloning by limiting dilution and selection in kanamycin- and gentamicin-supplemented medium. Clones were PCR-screened using primers for kanamycin and gentamicin-resistance cassettes (B70, B71, B348, B349), as well as BBK32 and Tp0751 expression cassettes (B1723, B1724). Screening results for Tp0751-expressing *B*. *burgdorferi* clones are shown in S2 Fig. BBK32- and Tp0751-expressing *B*. *burgdorferi* clones used in subsequent experiments were TMB103 and TMB49. The sequence of the Tp0751 expression cassette in TMB49 was confirmed by sequencing of DNA isolated from this strain. No notable differences in bacterial morphology, motility or length were observed for any of the *B*. *burgdorferi* strains generated (S5 Table), suggesting that the presence of the *tp0751* coding sequence did not markedly affect *B*. *burgdorferi* viability.

### gDNA isolation

The *Bb*-Tp0751 strain was grown to logarithmic phase (5 x 10^7^ cells/ml) in 15 ml of BSK-II. Cells were spun down at 4,000 x g/15 min at 4°C, and frozen at -20°C to enhance cell lysis. Genomic DNA was isolated using PureLink Genomic DNA Mini Kit (Invitrogen, Burlington, ON, CA) according to manufacturer’s protocol for DNA isolation from Gram-negative bacteria with the following modifications. Thawed cells were resuspended in 180 μl of Genomic digestion buffer supplemented with 20μl of proteinase K, and incubated for 2 hours at 55°C with slow shaking. Genomic DNA was eluted using 50 μl of PCR grade water pre-warmed to 55°C.

### Sequencing

One microliter of isolated *Bb*-Tp0751 gDNA was used as a template for PCR. The reaction mixture contained 1 μL of 10 mM dNTPs (ThermoFisher Scientific, Toronto, ON), 1 μL of 25 μM F_Xho_Pfla_short, 1μL of 25μM Bb-751_int_R, 10 μL 5x Phusion HF buffer, 0.5 μL Phusion polymerase (both New England Biolabs (NEB), Whitby, ON), 1.5 μl of 100% DMSO and PCR grade water in 50 μL volume. PCR conditions were set as follows: 98°C/3min, 35 cycles (98°C/15 s, 58°C/30 s and 72°C/1 min) and 72°C/7 min. PCR products were purified using Qiaquick kit (QIAgen, Montréal, QC) according to manufacturer’s instructions, and eluted using 30 μL of provided elution buffer (pre-warmed to 55°C). Amplicons were Sanger sequenced using amplification primers at The Center for Applied Genomics at The Hospital for Sick Children (Toronto, ON). Each sequencing reaction contained 50–100 ng of PCR product and 7 pmol of the amplification primer (F_Xho_Pfla_short or Bb-751_int_R).

### RNA isolation

Biological triplicates and duplicates of the *Bb*-Tp0751 and the parent strain GCB706, respectively, were grown to logarithmic phase (5 x 10^7^ cells/ml) in 15 ml of BSK-II. Cells were harvested at 4,000 x g/15 min at 4°C, resuspended in 1 ml of Trizol reagent (Life Technologies), and placed at -80°C until processing. RNA was isolated according to the manufacturer’s instructions under RNase free conditions, and eluted using 30 μl of Ultrapure DNase/RNase-free water (Life Technologies). Average RNA yield was 805 ng/μl and 465 ng/μl for *Bb*-Tp0751 and the parent strain, respectively. Ten micrograms of RNA were treated with DNase using the Turbo DNA-free kit (Life Technologies) according to the manufacturers’ instructions. Briefly, the reaction was set up in a total volume of 50 μL with 1 μl of DNase. Samples were incubated at 37°C for 30 min, an additional 1 μl of DNase was added, and the incubation was continued for another 30 min at 37°C. DNase was inactivated using reagents provided in the kit. Integrity of RNA after DNase treatment was verified on an agarose gel. After DNase treatment, average RNA concentrations were 127.7 ng/μl (A260/280: 2.03–2.08) and 161.7 ng/μl (A260/280: 2.02–2.05) for the *Bb*-Tp0751 and the parent strain, respectively, as measured using a Nanodrop.

### cDNA preparation and digital PCR

Five microliters of isolated RNA were used as a template for reverse transcription PCR (RT-PCR), which was performed using the iSCRIPT cDNA synthesis kit (BioRad, Mississauga, ON) according to the manufacturer’s protocol. The *Bb*-Tp0751 RNA samples were pooled, and 5 μl were used as a template for no reverse transcriptase control (NRT) reaction. Three microliters of the RT-PCR sample was used as a template for digital PCR (10 μl of QX200 ddPCR EvaGreen Supermix (Biorad), 0.5 μl of 10 mM qTp0751FI, 0.5 μl of 10 mM qTp0751RI and PCR grade water in a 20 μl volume). Droplets were generated on the QX200 Droplet Generator (BioRad) and transferred to a 96-well plate for amplification. PCR conditions were set as follows with ramp rate 2°C/s: 95°C/5 min, 40 cycles (95°C/30 s. 58°C/1 min 30 s), 4°C/5 min, 90°C/5 min, and 4°C/5 min. Fluorescence measurements were performed using QX200 Droplet Reader (BioRad). Results were normalized to 100 ng of mRNA used for RT-PCR (S4 Table).

### Fluorescence-activated cell sorting (FACS) measurement of surface protein expression

Flow cytometry to measure expression and surface-localization of 3 XFLAG-tagged adhesins was performed as described with 1 x 10^8^ *B*. *burgdorferi*, using 1:250 dilutions of mouse anti-Flag-M2, mouse anti-FlaB and Alexa635-conjugated goat anti-mouse antibodies (Sigma, Invitrogen) [65]. After fixation in formalin, FACS analysis of equal numbers of bacteria was performed within 2 days of sample preparation using a Becton Dickinson FACSCalibur flow cytometer equipped with a 15 mW 488 nm argon laser, standard three-color filters, and CELLQuest Software (BD Bioscience, Franklin Lakes, NJ). Mean fluorescence intensities (MFI) of spirochetes obtained by analysis using FlowJo software (Three star Inc, Ashland, OR) were used to calculate relative levels of protein production in different strains, which were all normalized to values for the FLAG-tagged BBK32-expressing strain. Spirochetes with MFIs of less than 10 were considered negative for FLAG-tagged proteins.

### Peptide competitive inhibition assay: cell culturing and manipulation

Human umbilical vein endothelial cells (HUVECs) pooled from multiple donors and purchased from Lonza (Allendale, NJ) were cultured in endothelial growth medium-2 (EGM-2) (Lonza) at 37°C in an atmosphere of 5% CO_2_, as per manufacturer’s instructions. *B*. *burgdorferi* strains were cultivated at 36°C in an atmosphere of 1.5% CO_2_ in BSK-II medium [66] prepared in-house with appropriate antibiotics (100 μg/mL gentamycin, 200 μg/mL kanamycin).

### Peptide competitive inhibition assay: Stationary adhesion assay

Stationary adhesion assays were adapted from Szczepanski and colleagues [67] and Cameron and colleagues [19]. HUVECs (passage 2–3) were seeded in 4-well chamber slides (Nalge Nunc International, Rochester, NY), coated with 500 μg/mL phenol red-free matrigel (Corning, Tewksbury, MA) and grown 20 h at 37°C in 5% CO_2_ to form confluent monolayers. Synthetic Tp0751 peptides (p4, p6, p10, p11, p8, p4scr, p6scr, and p10scr1) were diluted to 500 μg/ml in HEPES buffered saline solution (Lonza) and HUVECs were pre-incubated with 0.1 μg of Tp0751 peptide per well for 3 h at 37°C in 5% CO_2_. Peptide solutions were removed from HUVECs prior to the addition of bacteria. *Borrelia burgdorferi* strains were cultured in biological triplicate, and two days prior to the experiment, *B*. *burgdorferi* cultures were passaged to 6x10^5^ cells/ml and grown for 48 h to reach a concentration of 2x10^7^ cells/ml. *Borrelia burgdorferi* cultures were then centrifuged and resuspended in a 3:1 mixture of BSK-II:EGM-2 and 1.4x10^7^ cells of each biological replicate were added to HUVECs in duplicate wells. Chamber slides were incubated for 12 h at 36°C in 1.5% CO_2_, washed three times with warm HEPES buffered saline to remove non-adherent bacteria, and fixed in buffered 10% formalin (Fischer Scientific, Ottawa, ON). To determine the effect of increasing concentrations of p10 and a negative control peptide (p8) on adherence of Parent and *Bb*-Tp0751 to HUVECs, cell binding assays were performed as above with cells preincubated with increasing concentrations of peptide (0 nM, 0.54 nM, 5.45 nM, 54.5 nM, and 545 nM). Quantitation of *B*. *burgdorferi* adhesion to HUVECs was performed by counting GFP-expressing bacteria in ten fields of view (FOV) from duplicate wells for each biological replicate under 400X magnification on a Nikon 80i fluorescence microscope (Meridian Instrument Company, Inc., Kent, WA) fitted with a monochrome digital camera, a dark-field condenser, and fluorescein filter (Excelitas Technologies, Mississauga, ON). Statistical analyses were performed using the Student’s two-tailed *t*-test.

## Supporting Information

S1 Fig Dose dependent binding of TP0751_78A and Tp0751_78 WT to fibrinogen. Dose-dependent binding assays were performed to evaluate the level of binding of Tp0751_78A used for crystallization and Tp0751_78 WT to fibrinogen (Fg). Average absorbance readings (600 nm) from three wells are presented with bars indicating standard error and the results are representative of two independent experiments. Apparent *K*_d_ values calculated using GraphPad Prism: Tp0751_78A = 2.0 ± 0.4 μM (Fg); wild-type Tp0751_78 WT = 6.1 ± 1.7 μM (Fg).
(PDF)

S2 Fig PCR screening of Tp0751-expressing *B*. *burgdorferi*. All PCR reactions were performed on DNA extracted from the same strains. “N.T.” designates the no template control lane (note the “N.T.” samples were all run on a separate gel and were spliced onto the images). “pCC_3–1” denotes the positive control reactions performed with the *E*. *coli*-derived plasmid control. (A) A 739 bp fragment of the kanamycin-resistance gene on the pCE320-derived shuttle vector carrying inserts for Tp0751 expression. (B) A 300 bp fragment of Tp0751-encoding inserts in the shuttle vector. (C) A 450 bp portion of the gentamicin resistance gene carried by the pTM61 plasmid encoding GFP. DNA ladder: 100 bp ladder (GeneRuler 100bp Plus DNA ladder, Fermentas).
(PDF)

S3 Fig Copy numbers of Tp0751 mRNA, as measured by digital PCR. Copy numbers were normalized to 100 ng of input mRNA used for RT-PCR. Dots stand for individual biological replicates. Bars represent means; black bar corresponds to measured copy numbers, full and dotted grey lines correspond to Poisson-corrected values (maximum and minimum, respectively), as determined by QuantaSoft software (BioRad). NRT—no reverse transcriptase control for pooled *Bb*-Tp0751 mRNA samples.
(PDF)

S1 Table *Borrelia burgdorferi* strains. (PDF)

S2 Table Plasmid constructs. (PDF)

S3 Table Primers. (PDF)

S4 Table Digital PCR summary. (PDF)

S5 Table Bacterial length of individual *Borrelia burgdorferi* strains. (PDF)
