# Supplementary figures and images for: The Structure of Treponema pallidum Tp0751 (Pallilysin) Reveals a Non-canonical Lipocalin Fold That Mediates Adhesion to Extracellular Matrix Components and Interactions with Host Cells
Source: PLoS Pathog. 2016 Sep 28;12(9):e1005919. doi: 10.1371/journal.ppat.1005919 (PMC5040251; doi:10.1371/journal.ppat.1005919)

**S1 Fig. Dose dependent binding of TP0751\_78A and Tp0751\_78 WT to fibrinogen.**

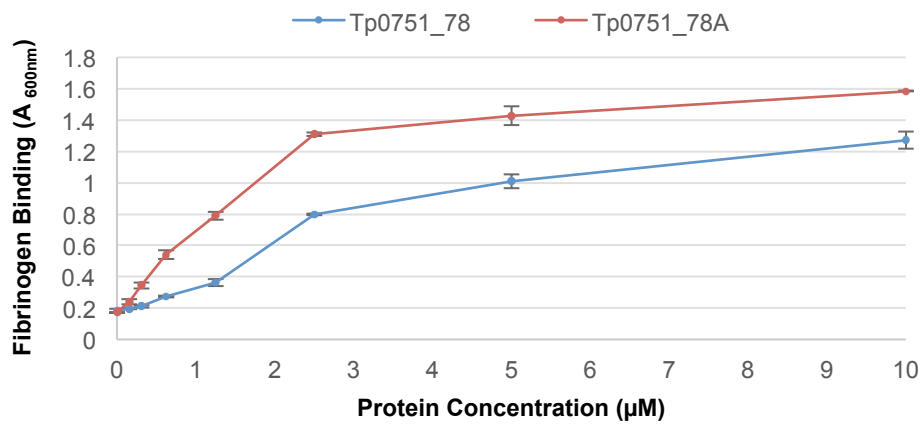

Supplement: S1 Fig — Dose-dependent binding assays were performed to evaluate the level of binding of Tp0751_78A used for crystallization and Tp0751_78 WT to fibrinogen (Fg). Average absorbance readings (600 nm) from three wells are presented with bars indicating standard error and the results are representative of two independent experiments. Apparent K d values calculated using GraphPad Prism: Tp0751_78A = 2.0 ± 0.4 μM (Fg); wild-type Tp0751_78 WT = 6.1 ± 1.7 μM (Fg). (PDF) [file ppat.1005919.s001.pdf]

**S2 Fig. PCR screening of Tp0751-expressing *B. burgdorferi*.**

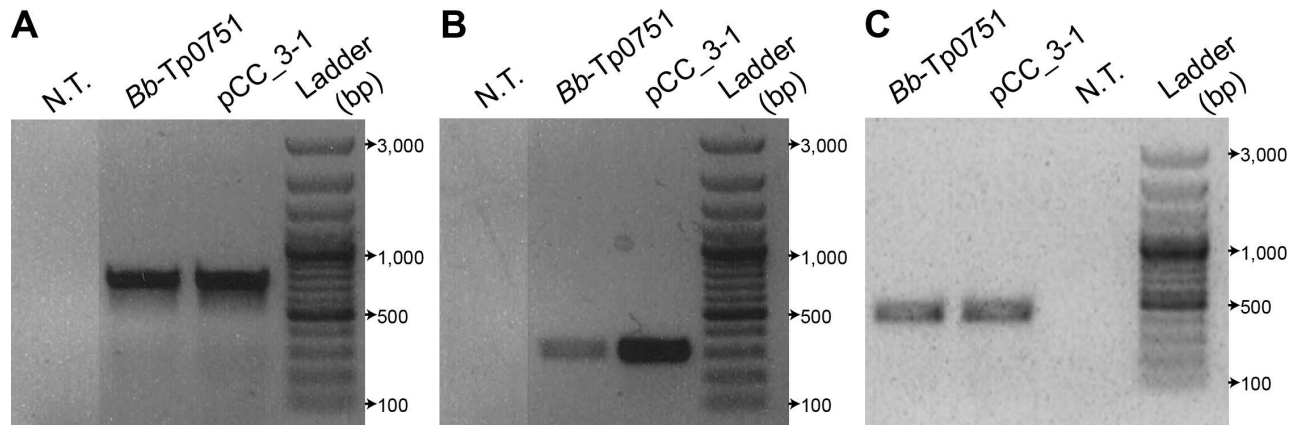

Supplement: S2 Fig — All PCR reactions were performed on DNA extracted from the same strains. “N.T.” designates the no template control lane (note the “N.T.” samples were all run on a separate gel and were spliced onto the images). “pCC_3–1” denotes the positive control reactions performed with the E. coli-derived plasmid control. (A) A 739 bp fragment of the kanamycin-resistance gene on the pCE320-derived shuttle vector carrying inserts for Tp0751 expression. (B) A 300 bp fragment of Tp0751-encoding inserts in the shuttle vector. (C) A 450 bp portion of the gentamicin resistance gene carried by the pTM61 plasmid encoding GFP. DNA ladder: 100 bp ladder (GeneRuler 100bp Plus DNA ladder, Fermentas). (PDF) [file ppat.1005919.s002.pdf]

**S3 Fig. Copy numbers of Tp0751 mRNA, as measured by digital PCR.**

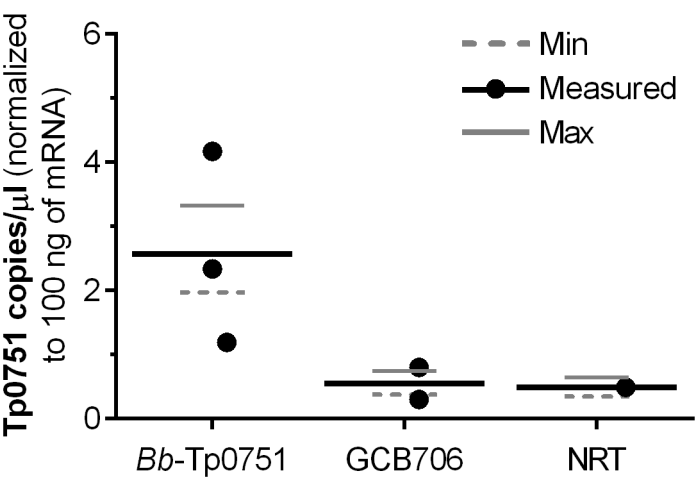

Supplement: S3 Fig — Copy numbers were normalized to 100 ng of input mRNA used for RT-PCR. Dots stand for individual biological replicates. Bars represent means; black bar corresponds to measured copy numbers, full and dotted grey lines correspond to Poisson-corrected values (maximum and minimum, respectively), as determined by QuantaSoft software (BioRad). NRT—no reverse transcriptase control for pooled Bb-Tp0751 mRNA samples. (PDF) [file ppat.1005919.s003.pdf]
