# Supplementary material for: The Structure of Treponema pallidum Tp0751 (Pallilysin) Reveals a Non-canonical Lipocalin Fold That Mediates Adhesion to Extracellular Matrix Components and Interactions with Host Cells
Source: PLoS Pathog. 2016 Sep 28;12(9):e1005919. doi: 10.1371/journal.ppat.1005919 (PMC5040251; doi:10.1371/journal.ppat.1005919)
Supplement: S1 Table — (PDF) [file ppat.1005919.s004.pdf]

S1 Table. ***B. burgdorferi*** **strains**

Strain number	Background	Description	Antibiotic resistance	Reference
GCB706	B31-A	“Parent”: Non-infectious high passage GFP-expressing strain	gent	[63]
TMB103	GCB706	GCB706 transformed with pTM259 expressing BBK32-3XFLAG	gent, kan	This study
TMB48	GCB706	GCB706 transformed with pCC_3-1 expressing Tp0751-3XFLAG	gent, kan	This study
