# Supplementary material for: The Structure of Treponema pallidum Tp0751 (Pallilysin) Reveals a Non-canonical Lipocalin Fold That Mediates Adhesion to Extracellular Matrix Components and Interactions with Host Cells
Source: PLoS Pathog. 2016 Sep 28;12(9):e1005919. doi: 10.1371/journal.ppat.1005919 (PMC5040251; doi:10.1371/journal.ppat.1005919)
Supplement: S2 Table — (PDF) [file ppat.1005919.s005.pdf]

S2 Table. Plasmid constructs

Plasmid	*E. coli* strain	Description	Reference
pTM259	GCE1882	***PflaB*-driven BBK32-3XFLAG expression** cassette cloned into pCE320 [50] via *Xho*I/*Not*I; *PflaB* sequence amplified from pTM61 [63]; *bbk32* signal + coding sequences amplified from pBBK32 [68]; C-terminal *3XFLAG* coding sequence amplified from pJL148SPA [69]. Primers for cloning: B1723, B1662, B1663, B1724, B1726, B1727.	This study
pCC_3-1	TME390	***PflaB*-driven Tp0751-3XFLAG expression** cassette cloned into pTM259 via *Xho*I/*Not*I; *PflaB* sequence amplified from pTM259; *tp0751* signal + coding sequences amplified from pENTR clone 16 [23]; C-terminal *3XFLAG* coding sequence amplified from pTM259. Primers used for cloning: B1723, B1724, P2, P3, P7, P8	This study
