# Supplementary material for: The Structure of Treponema pallidum Tp0751 (Pallilysin) Reveals a Non-canonical Lipocalin Fold That Mediates Adhesion to Extracellular Matrix Components and Interactions with Host Cells
Source: PLoS Pathog. 2016 Sep 28;12(9):e1005919. doi: 10.1371/journal.ppat.1005919 (PMC5040251; doi:10.1371/journal.ppat.1005919)
Supplement: S3 Table — (PDF) [file ppat.1005919.s006.pdf]

S3 Table. Primers

Primer	Target	Sequence (5'-3')
B1723 (P1)	5' end of *PflaB* (with *Xho*I site) (forward primer)	CTCGAGTGTCTGTCGCCTCTTGTGG
B1662	3' end of *PflaB* fused to 5' end of *bbk32* coding sequence (reverse primer)	CAAATATTTACTTTTAACTTTTTTCATTCATTCCTCCATGATAAAATTTAA
B1663	3' end of *PflaB* fused to 5' end of *bbk32* coding sequence (forward primer)	TTAAATTTTATCATGGAGGAATGAATGAAAAAAGTTAAAAGTAAATATTTG
B1727	3' end of *bbk32* coding sequence fused to 5' end of *3XFLAG* coding sequence (reverse primer)	CCGTCATGGTCTTTGTAGTCGTACCAAACGCCATTCTTGTC
B1726	3' end of *bbk32* coding sequence fused to 5' end of *3XFLAG* coding sequence (forward primer)	GACAAGAATGGCGTTTGGTACGACTACAAAGACCATGACGG
B1724 (P9)	3' end of *3XFLAG* coding sequence (with *Not*I site) (reverse primer)	GCGGCCGCCTACTTGTCATCGTCATCCTTGTA
P2	3' end of *PflaB* fused to 5' end of *tp0751* coding sequence (forward primer)	TTAAATTTTATCATGGAGGAATGAGTGAATAGACCTTTACTGAGTG
P3	3' end of *PflaB* fused to 5' end of *tp0751* coding sequence (reverse primer)	CACTCAGTAAAGGTCTATTCACTCATTCCTCCATGATAAAATTTAA
P7	3' end of *tp0751* coding sequence fused to 5' end of *3XFLAG* (forward primer)	GCTAGTGCTCCTTCGCCCGACTACAAAGACCATGACGG
P8	3' end of *tp0751* coding sequence fused to 5' end of *3XFLAG* (reverse primer)	CCGTCATGGTCTTTGTAGTCGGGCGAAGGAGCACTAGC
B70	Forward primer: screening for *kan*^{R} gene	CATATGAGCCATATTCAACGGGAAACG
B71	Reverse primer: screening for *kan*^{R} gene	AAAGCCGTTTCTGTAATGAAGGAG
B348	Forward primer: screening for *gent*^{R} gene	CGCAGCAGCAACGATGTTAC
B349	Reverse primer: screening for *gent*^{R} gene	CTTGCACGTAGATCACATAAGC
pJETSEQF	Forward primer for pJET1.2 insert sequencing	CGACTCACTATAGGGAGAGCGGC
pJETSEQR	Reverse primer for pJET1.2 insert sequencing	AAGAACATCGATTTTCCATGGCAG
F_Xho_Pfla_short	Forward primer for *Bb*-Tp0751 construct sequencing	CTCGAGTGTCTGTCGCCTC
Bb-751_int_R	Reverse primer for *Bb*-Tp0751 construct sequencing	CCGTGCTACGTCTTCAACCG
qTp0751FI	Forward primer for digital PCR (*Bb*-Tp0751)	GTCTGACACGGCAGACAATA
qTp0751RI	Reverse primer for digital PCR (*Bb*-Tp0751)	GTTGTGTGTGTGTCTGTGCT
