# Supplementary material for: The Structure of Treponema pallidum Tp0751 (Pallilysin) Reveals a Non-canonical Lipocalin Fold That Mediates Adhesion to Extracellular Matrix Components and Interactions with Host Cells
Source: PLoS Pathog. 2016 Sep 28;12(9):e1005919. doi: 10.1371/journal.ppat.1005919 (PMC5040251; doi:10.1371/journal.ppat.1005919)
Supplement: S4 Table — (PDF) [file ppat.1005919.s007.pdf]

S4 Table: Digital PCR result summary

	Concentration (copies/µl) Raw data			Concentration (copies/µl) Normalized to 100 ng of input RNA		
	Measured	Poisson corrected min	Poisson corrected max	Measured	Poisson corrected min	Poisson corrected max
TMB49-1	6.00	7.50	4.70	4.17	5.21	3.27
TMB49-2	1.14	1.62	0.84	1.19	1.69	0.88
TMB49-3	2.80	3.70	2.10	2.34	3.09	1.76
706-1	0.24	0.39	0.14	0.30	0.48	0.17
706-2	0.90	1.14	0.66	0.80	1.01	0.59
NRT (TMB49)	0.63	0.84	0.45	0.49	0.65	0.35
NT	0.28	0.44	0.16	NA	NA	NA

Note: For low copy number samples, the negative threshold (indicated at the bottom of the table) is usually set at 3*values for the average negative control value. Negative controls are highlighted in red. NRT – no reverse transcriptase control, NT – no template control and NA – not applicable.
