# Supplementary material for: The Structure of Treponema pallidum Tp0751 (Pallilysin) Reveals a Non-canonical Lipocalin Fold That Mediates Adhesion to Extracellular Matrix Components and Interactions with Host Cells
Source: PLoS Pathog. 2016 Sep 28;12(9):e1005919. doi: 10.1371/journal.ppat.1005919 (PMC5040251; doi:10.1371/journal.ppat.1005919)
Supplement: S5 Table — (PDF) [file ppat.1005919.s008.pdf]

S5 Table. Bacterial length of individual *B. burgdorferi* strains

**Strain**	**Bacterial length (µm) ±SEM**
Parent GCB706	16.34 ± 4.068
Parent+BBK32 (TMB103)	18.09 ± 3.597
Parent+*Tp*-Tp0751 (TMB49)	17.52 ± 4.498
